# Phytochemical Analysis and Antimalarial Activity Aqueous Extract of *Lecaniodiscus cupanioides Root*

**DOI:** 10.1155/2013/605393

**Published:** 2013-08-01

**Authors:** Mikhail Olugbemiro Nafiu, Taoheed Adedeji Abdulsalam, Musbau Adewumi Akanji

**Affiliations:** Department of Biochemistry, University of Ilorin, P. M. B. 1515 Ilorin, Nigeria

## Abstract

Root aqueous extract of *Lecaniodiscus cupanioides* was evaluated for antimalarial activity and analyzed for its phytochemical constituents. Twenty-four (24) albino mice were infected by intraperitoneal injection of standard inoculum of chloroquine sensitive *Plasmodium berghei* (NK 65). The animals were randomly divided into 6 groups of 3 mice each. Group 1 served as the control while groups II–IV were orally administered 50, 150, and 250 mg/kg body weights of extract. Groups 5 and 6 received 1.75 and 5 mg/kg of artesunate and chloroquine, respectively. The results of the phytochemical analysis showed the presence of alkaloids (2.37%), saponin (0.336), tannin (0.012 per cent), phenol (0.008 per cent), and anthraquinone (0.002 per cent). There was 100 per cent parasite inhibition in the chloroquine group and 70 per cent in the 50 mg/kg body weight on day 12, respectively. The mean survival time (MST), for the control group was 14 days, artesunate 16 days, and chloroquine 30 days, while the groups that received 50 and 250 mg/kg body weight recorded similar MST of 17 days and the 150 mg/kg body weight group recorded 19 days. The results obtained indicated that the aqueous extract of *Lecaniodiscus cupanioides* may provide an alternative antimalarial.

## 1. Introduction

Malaria is an enormous health, social, and economic burden for over 40% of the world's population. It remains one of the most important infectious diseases of mankind, killing 1–3 million people and causing morbidity in more than 500 million people annually [1]. Almost 90 per cent of the deaths from malaria occur in sub-Saharan Africa, where the vulnerable groups are children under 5 years and the pregnant women [2]. 

The control of malaria is hampered by the rapid selection of parasites resistant to antimalarials. Indeed, there is no single antimalarial in clinical use to which the parasite has not yet developed resistance [3, 4]. Antimalaria drug resistance has become one of the greatest challenges against malaria control. There is widespread multidrug resistance to common antimalarial drugs [1, 5].

Rodent plasmodia such as *Plasmodium berghei *are commonly used as malaria models in mice and have tremendous impact on the investigations of antimalarial activities of plant extracts. The need to search and develop more effective antimalarial drugs that are inexpensive and readily available to people in the developing countries like Nigeria has necessitated this study. Medicinal plants have been the focus of many anti-infective drugs and alternative sources of antimalarial agents in various parts of the world since long ago [6]. One of such plants claimed to possess antimalarial activities is root of *Lecaniodiscus cupanioides.*

*Lecaniodiscus cupanioides* is a tropical plant widely distributed in Africa and Asia. It belongs to the Sapindaceae family and it is identified by various names in Nigeria, such as Ukpo (Igbo), Utantan (Edo), Kafi-nama-zaki (Hausa), and Akika (Yoruba). The plant is ethnomedically reputed to be useful in the treatment of wounds and sores, abdominal swelling caused by liver abscess, fevers, measles, hepatomegaly and burns, among others [7]. The present study was carried out to assess the antimalarial activities of the aqueous extract of root of *Lecaniodiscus cupanioides *in mice.

## 2. Materials and Methods

### 2.1. Plant Materials

The root of *Lecaniodiscus cupanioides *(Sapindaceae) was obtained from “Oja-Oba,” Ilorin, Kwara state, and was identified in the Department of Plant Biology, University of Ilorin, Ilorin, Kwara state, Nigeria

### 2.2. Aqueous Extraction

The root of the plant was air-dried to constant weight and ground into powdered form with an electric milling machine. Aqueous extract was prepared by soaking 200 g of the powered root in 1.2 liters of distilled water for 24 hours in amber colored bottles. The contents of the bottles were filtered with Whatman no. 1 filter paper and the filtrate collected was evaporated to dryness with steam on water bath (40°C). The dried extract was weighed and reconstituted into desired doses.

### 2.3. Phytochemical Analysis

A portion of the root powder was subjected to phytochemical analysis, using standard chemical test as described by [8, 9].

### 2.4. Experimental Animals

Albino mice with average weight of 21.1 g were obtained from the small Animal Holding Unit, Department of Biochemistry, University of Ilorin, Ilorin, Nigeria. The animals were housed in well ventilated plastic cages under standard conditions, and the study was conducted in accordance with the recommendations from the declaration of Helsinki on guiding principles in the care and use of animals.

### 2.5. Drugs and Reagents

Artesunate used in this study was obtained from Mekophar Chemical Pharmaceutical Joint Stock Company, Vietnam, while chloroquine was from May and Baker Pharmaceutical Company Limited, Nigeria. Other reagents were of analytical grade and were prepared in all glass bottles.

### 2.6. Malaria Parasite

*Plasmodium berghei* (chloroquine sensitive NK65 strain) was obtained from the Institute for Advanced Medical Research and Training (IMRAT), College of Medicine, University of Ibadan, Ibadan Nigeria.

### 2.7. Inoculation of Experimental Mice

Albino mice were infected by intraperitoneal injection of standard inoculum (0.2 mL of 1 × 10^7^ infected erythrocytes) from a single donor mouse previously infected with *Plasmodium berghei* (29 per cent parasitemia). 

### 2.8. Animal Groupings

The animals were randomly divided into 6 groups of 3 mice each, after confirmation of parasitaemia 72 hours after infection. Group 1 (control) was left untreated but administered appropriate volume of distilled water. Groups 2, 3, and 4 were administered aqueous extract of *Lecaniodiscus cupanioides* through oropharyngeal canulla at the doses of 50, 150, and 250 mg/kg body weight, respectively. Group 5 received artesunate at a dose of 1.75 mg/kg body weight daily for 3 days while Group 6 animals were treated with 5 mg/kg body weight of chloroquine for the same period. 

### 2.9. Estimation of Percentage Parasitaemia

Percentage parasitemia was estimated at the end of the observational period of 28 days using the formula

(1) $$\begin{array}{c} \frac{\text{parasitized RBC}}{\text{parasitized RBC}+\text{nonparasitized RBC}}\times 100. \end{array}$$

### 2.10. Estimation of Percentage Chemosuppression

The percentage chemo-suppression of parasite multiplication per days was calculated by using the formula

(2) $$\begin{array}{c} \%\;\text{chemo-suppression}\;A=\;\frac{B-C}{C}\times 100, \end{array}$$

where *B* = parasitaemia in study group, *C* = parasitaemia in control.

### 2.11. Estimation of Mean Survival Time (MST)

The number of days each animal survived was recorded for the animals in each group and mean survival time calculated using the formula

(3) $$\begin{array}{c} \text{MST}=\frac{\text{sum of days of survival of animals/group}}{\text{total number of animals in the group}}. \end{array}$$

### 2.12. Statistical Analysis

Data were presented as mean of three determinations ± SEM. Statistical analysis was carried out using one-way analysis of variance (ANOVA). Differences were considered statistically significant at *P* < 0.05.

## 3. Results

Qualitative and quantitative screening of the components of the plant root revealed the phytochemicals shown in Table 1. The phytochemicals that were present included Alkaloids, saponin, tannins, phenol and anthraquinone.

### 3.1. Percentage Parasitaemia

**Figure 1:**
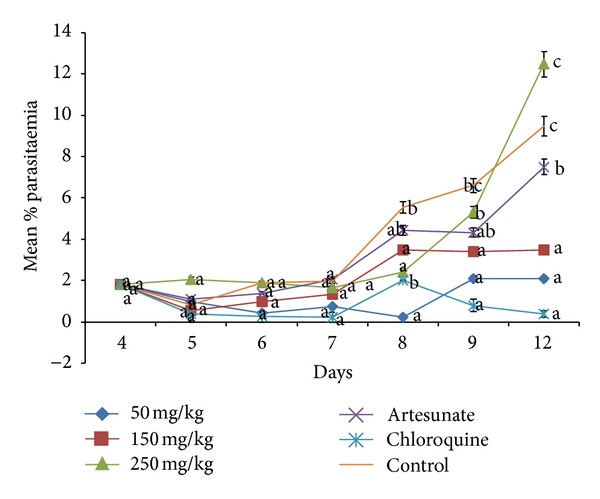
Parasitaemia changes following treatment with different doses of aqueous extract of *Lecaniodiscus cupanioides* and standard drugs. Values carrying different superscript from the control are significantly different (*P* < 0.05).

Estimation of percentage parasitemia at the end of 12 days showed the results in Figure 1. The 50 mg/kg body weight of the extract prevented further increase in the baseline parasitaemia until after day 8 before a noticeable rise in parasitaemia was observed. The 150 and 250 mg/kg body weights of the extract also initially reduced the parasitaemia baseline parasitaemia at day 5 but could not maintain the reduction compared to the 50 mg/kg body weight. A reduction in baseline parasitaemia was observed in the artesunate treated group before resurgence was in the level on day 6. Chloroquine reduced the parasitaemia level throughout the experimental days.

### 3.2. Mean Survival Time (MST)

Table 2 showed the mean survival time for the animals in each group. The least MST of 14 days was recorded for the control group that was left untreated. The mice in the artesunate group recorded MST of 16 days. The MST of 17 days was recorded for the groups that received 50 and 250 mg/kg while 19 days were recorded for the group that received 150 mg/kg body weight of the extract. MST of 30 days was recorded for chloroquine.

## 4. Discussion

The phytochemical analysis of the aqueous root extract of *Lecaniodiscus cupanioides* revealed the presence of alkaloids, saponin tannin, phenol, and anthraquinone. Alkaloids have been implicated in antimalarial activity of many plants [10]. The extract of *Nigella sativa* (black seed) contained different classes of alkaloids that were believed to block protein synthesis in *Plasmodium falciparum* [11]. Triterpenoid and steroid saponins have been found to be detrimental to several infectious protozoans such as *Plasmodium falciparum* [12].

Although primate models provide a better prediction of efficacy in humans than the rodent models, the latter have also been validated through the identification of several conventional antimalarials, such as chloroquine, halofantrine, mefloquine, and more recently artemisinin derivatives [13]. *P. berghei* are used in the prediction of treatment outcomes. Hence it was an appropriate parasite for the study. As this parasite is sensitive to chloroquine, this drug was employed as a standard drug in this study.

Rane test is a standard test commonly used for antimalarial screening. It relies on the ability of standard inoculum of *P. berghei* to kill the recipient mouse thereafter. The extension of survival by the test compound beyond 12 days is regarded as active [14].

When a standard antimalarial drug is used in mice infected with *P. berghei*, it suppresses parasitaemia to nondetectable level [15], which is in agreement with the effect of chloroquine in this study. Results from this study showed that the aqueous root extract of *L. cupanioides* possesses antimalarial activity that was comparable to that of chloroquine. The observed antimalarial activity was not dose dependent but at a dose of 50 mg/ kg body weight a reduction in parasitaemia was recorded with 70 per cent chemo-suppression on day 12 (Table 4). It was also reported that potent antimalarial activities exhibited by *Eurycoma longifolia* with 80% parasitaemia inhibition at 10 mg/kg and *Ardisia crispa* [16]. The high parasitaemia obtained at 150 and 250 mg/kg of the extract compared to 50 mg/kg after the fifth day (Figure 1) may mean that at higher doses the extract exacerbated the infection. This may be due to immunosuppressive activity of the extract. It had been reported that oral administration of phytochemicals like saponin, tannins, and phenols possessed ability to suppress cellular immunity [17]. 

The determination of percentage inhibition of parasitaemia is the most reliable parameter in antimalarial screening. A mean group parasitaemia level of less than or equal to 90 per cent of that of treated control animals usually indicates that the test compound is active in standard screening studies [14]. Therefore, it is clear from the result (Table 3) that the *P. berghei*–infected mice treated with the aqueous root extract of *L. cupanioides* reduced in percentage parasitaemia compared to those of the untreated control animals. The possible active compounds responsible for the antimalarial activity of *L. cupanioides* may be alkaloid and saponin. The results from this study, therefore, lent scientific credence to the folkloric use of the root extract of *L. cupanioides* for the treatment of malaria in Africa.

## 5. Conclusion

The present study revealed that oral administration of aqueous root extract of *L. cupanioides* exhibited antimalarial activity in *P. berghei*–infected mice. This suggests the beneficial effects of the herb. It is, however, very important to note that the extract at 50 mg/kg body weight showed the best action. The effect of the extract compared favourably with chloroquine and artesunate. These findings have lent scientific support to the folkloric use of aqueous root extract of *L. cupanioides.*

## Figures and Tables

**Table 1:** Parasitaemia changes following treatment with different doses of aqueous extract of *Lecaniodiscus cupanioides* and standard drugs.

Treatments	Days						
	4	5	6	7	8	9	12
Control	1.80 ± 0.12^a^	0.83 ± 0.08^a^	1.90 ± 0.40^a^	1.97 ± 0.14^a^	5.53 ± 1.98^b^	6.60 ± 1.41^cb^	9.47 ± 1.18^c^
Chloroquine	1.80 ± 0.12^a^	0.40 ± 0.40^b^	0.27 ± 0.27^b^	0.23 ± 0.23^b^	0.20 ± 0.00^b^	0.80 ± 0.29^b^	0.40 ± 0.17^b^
Artesunate	1.80 ± 0.12^a^	1.10 ± 0.59^a^	1.40 ± 0.70^a^	2.07 ± 1.03^a^	4.43 ± 2.41^ab^	4.33 ± 0.53^ab^	7.50 ± 2.82^a^
50 mg/kg b.wt extract	1.80 ± 0.12^a^	1.00 ± 0.58^a^	0.43 ± 0.43^a^	0.73 ± 0.38^a^	0.23 ± 0.12^a^	2.10 ± 0.45^a^	2.10 ± 1.3^a^
150 mg/kg b.wt extract	1.80 ± 0.12^a^	0.53 ± 0.53^a^	1.00 ± 1.00^a^	1.33 ± 1.33^a^	3.50 ± 1.75^a^	3.40 ± 1.70^a^	3.50 ± 0.35^a^
250 mg/kg b.wt extract	1.80 ± 0.12^a^	2.07 ± 0.54^a^	1.90 ± 0.00^a^	1.67 ± 0.83^a^	2.40 ± 1.44^a^	5.30 ± 0.00^b^	12.47 ± 1.07^c^

Data are mean of 3 replicates ± SEM. Values carrying different superscripts from the control along the rows are significantly different (*P* < 0.05).

**Table 2:** Phytochemical constituents of the aqueous root extract of *Lecaniodiscus cupanioides*.

Phytochemicals	Percentage
Alkaloids	2.37
Saponin	0.336
Tannin	0.012
Phenol	0.008
Anthraquinone	0.002
Steroids	nd
Phlobatannin	nd
Terpenes	nd
Cardiac glycosides	nd
Flavonoids	nd

nd: not detected.

**Table 3:** Mean survival time of animals for the experiment.

Group	Mean survival time (days)
Control	14
50 mg/kg	17
150 mg/kg	19
250 mg/kg	17
Artesunate	16
Chloroquine	28

**Table 4:** Percentage chemosuppression of parasitaemia by the aqueous root extract of *L. cupanioides* and standard drugs.

	Days					
	% suppression					
Group	5	6	7	8	9	12
50 mg/kg	60	60	60	90	70	70
150 mg/kg	60	30	30	50	60	10
250 mg/kg	50	40	30	20	20	20
Artesunate	50	20	30	20	30	10
Chloroquine	70	90	90	60	90	100
